# Determination of B-Cell Epitopes in Patients with Celiac Disease: Peptide Microarrays

**DOI:** 10.1371/journal.pone.0147777

**Published:** 2016-01-29

**Authors:** Rok Seon Choung, Eric V. Marietta, Carol T. Van Dyke, Tricia L. Brantner, John Rajasekaran, Pankaj J. Pasricha, Tianhao Wang, Kang Bei, Karthik Krishna, Hari K. Krishnamurthy, Melissa R. Snyder, Vasanth Jayaraman, Joseph A. Murray

**Affiliations:** 1 Division of Gastroenterology and Hepatology, Mayo Clinic, Rochester, MN, United States of America; 2 Vibrant Sciences LLC, San Carlos, CA, United States of America; 3 Center for Neurogastroenterology, Johns Hopkins University, Baltimore, MD, United States of America; 4 Division of Clinical Biochemistry and Immunology, Mayo Clinic, Rochester, MN, United States of America; Baylor College of Medicine, UNITED STATES

## Abstract

**Background:**

Most antibodies recognize conformational or discontinuous epitopes that have a specific 3-dimensional shape; however, determination of discontinuous B-cell epitopes is a major challenge in bioscience. Moreover, the current methods for identifying peptide epitopes often involve laborious, high-cost peptide screening programs. Here, we present a novel microarray method for identifying discontinuous B-cell epitopes in celiac disease (CD) by using a silicon-based peptide array and computational methods.

**Methods:**

Using a novel silicon-based microarray platform with a multi-pillar chip, overlapping 12-mer peptide sequences of all native and deamidated gliadins, which are known to trigger CD, were synthesized in situ and used to identify peptide epitopes.

**Results:**

Using a computational algorithm that considered disease specificity of peptide sequences, 2 distinct epitope sets were identified. Further, by combining the most discriminative 3-mer gliadin sequences with randomly interpolated3- or 6-mer peptide sequences, novel discontinuous epitopes were identified and further optimized to maximize disease discrimination. The final discontinuous epitope sets were tested in a confirmatory cohort of CD patients and controls, yielding 99% sensitivity and 100% specificity.

**Conclusions:**

These novel sets of epitopes derived from gliadin have a high degree of accuracy in differentiating CD from controls, compared with standard serologic tests. The method of ultra-high-density peptide microarray described here would be broadly useful to develop high-fidelity diagnostic tests and explore pathogenesis.

## Introduction

Antibody detection is one of the main approaches for the diagnosis of many diseases, including autoimmune disorders, infectious diseases, and cancers.[1–3] Indeed, the development of antibody-based assays has been intensively pursued for the diagnosis and treatment of many diseases; however, only a small number of biomarkers have been identified as effective.[1, 4] With peptide arrays, the overlapping synthetic peptide approach has been used as an effective way to map the epitope specificity of antibodies.[5–7] This method is especially effective for identifying linear antigenic epitopes derived from known target proteins, but has been restricted by the expense and logistics of acquiring and handling large numbers of peptides. Recent advances in semiconductor methods and the generation of high-throughput peptide microarrays using a combination of lithography and biochemistry for peptide synthesis have opened the door to a new era in the identification of novel biomarkers of disease.[5, 8, 9] Here, we describe a novel method for silicon-based peptide microarray with computational algorithm to identify the discontinuous epitopes from native and modified gliadin peptides that glutamic acid was substituted for glutamine, which are highly reactive in patients with celiac disease (CD).

CD is an autoimmune disease of the gastrointestinal tract that is triggered by exposure to dietary gluten, which is a group of storage proteins from wheat, barley, and rye.[10–12] While the pathogenesis of CD appears to be mediated by T cells reactive to gluten,[13–15] the serologic diagnosis relies on the presence of self-reactive antibodies or antibodies to exogenous antigens. However, it is not clear how the pathogenic epitopes are recognized by B cells during the development of CD. This interaction between B and T lymphocytes, which may play a central role in causing tissue destruction, would be crucial to understanding of the mechanism of disease initiation and to development of better clinical tools. We hypothesized that enzymatic modification of gliadin peptides and subsequent discontinuous transformation lead to recognition of the host immune system. Thus, we aimed to describe the novel platform and technology that uses a high-volume manufacturing and computational process derived from semiconductor industrial design to identify highly predictive biomarkers in a common chronic immune disease.

## Materials and Methods

### Population

To discover a novel biomarker for the diagnosis of CD, sera were collected from 3 sources: a cohort that was part of a previous study (48 biopsy-proven CD cases and 50 controls), [2] a cohort from ARUP Laboratories (42 biopsy-proven CD cases and 29 controls), and a commercially obtained cohort (12 rheumatoid arthritis cases and 7 systemic lupus erythematosus cases). In addition, sera of a confirmatory cohort (306 CD cases and 1,590 controls), which was assembled in the previous study in a community,[16] were used for evaluating the diagnostic utility of identified or newly developed peptide sets from the exploratory cohort. The demographic characteristics of the exploratory and confirmatory cohorts can be found as Table 1. All samples were handled according to standard procedures and stored at -80°C. All samples were probed using 1:101 primary antibody dilution and 1:2,000 secondary antibody dilution and scanned on a Nikon total solution platform consisting of a Hamilton fluidics station and second-generation microarray fluorescence scanner.

**Table 1:** Clinical Characteristics of the study Population.

	N	Age, mean (Range)	Sex, male (%)	Sex, female (%)
Exploratory population				
Celiac disease	90	39.4 (19.5–60.2)	43%	57%
Rheumatoid arthritis	12	40.6 (20.1–68.8)	50%	50%
Systemic Lupus Erythematosus	7	34.5 (25.7–57.0)	43%	57%
Healthy controls	79	40.2 (19.7–63.3)	48%	52%
Confirmatory population				
Celiac disease	306	35.2 (18.1–49.9)	38%	62%
Rheumatoid arthritis	75	39.0 (20.1–56.7)	50%	50%
Systemic Lupus Erythematosus	40	36.5 (25.3–45.6)	38%	62%
Healthy controls	1475	35.3 (18.5–69.8)	34%	66%

#### Study Approval

This study was conducted according to the principles expressed in the Declaration of Helsinki. Ethical approval, including informed consent, was obtained from the Mayo Clinic Institutional Review Board. All informed consents were documented by a written consent form approved by the IRB and signed by the subject or the subject's legally authorized representative.

### Wafer Substrate Preparation

**Fig 1:**
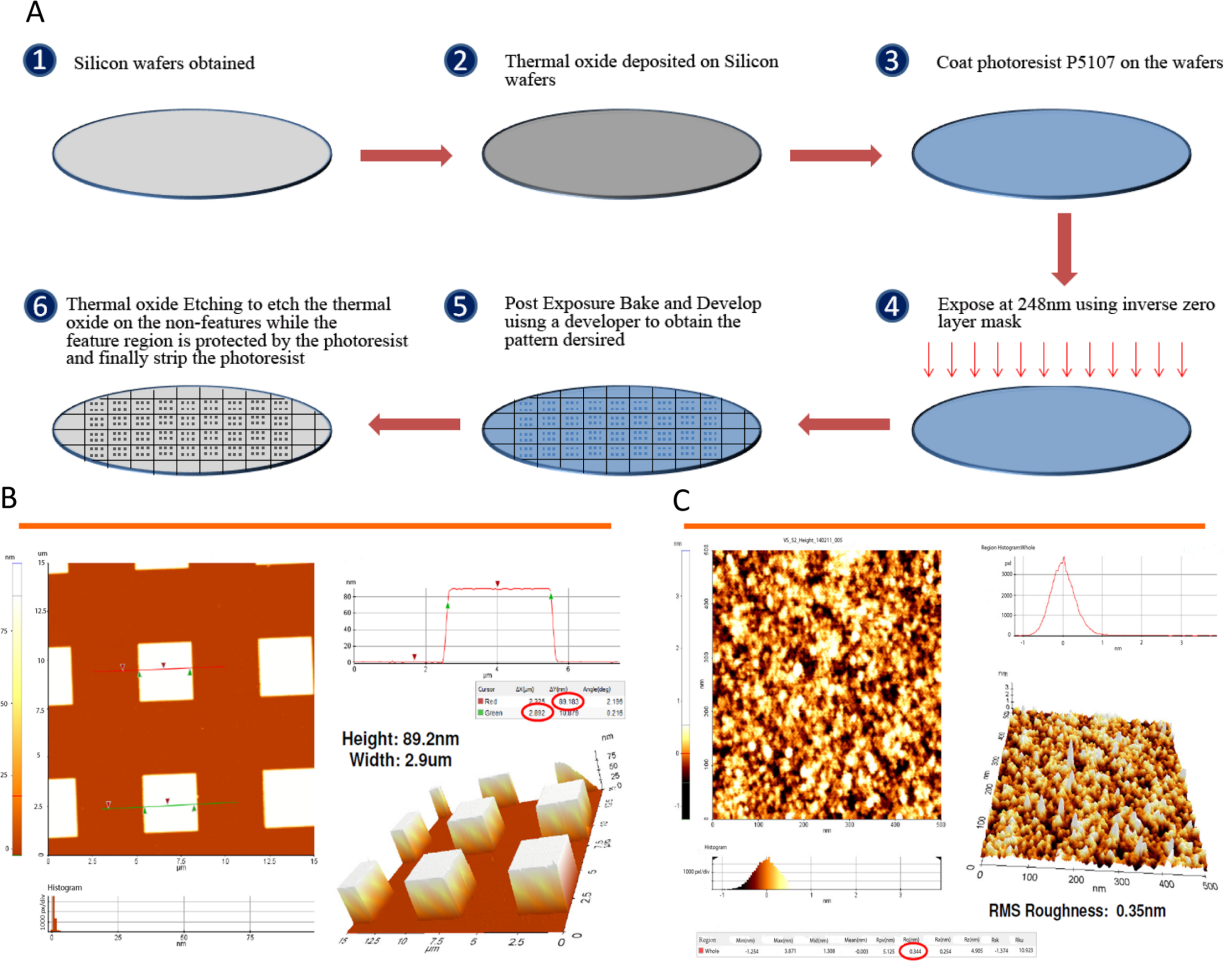
Silicon Wafer Processing. A) Silicon Wafer Substrate Preparation. 1) After silicon wafers obtained, 2) thermal oxide deposited on Silicon wafers. 3) Then, the wafer was uniformly coated with P5107 (photoresist, Rohm and Haas Chemicals), using a RF3S coater (Sokudo). 4) The wafer was exposed to 248nm wavelength ultraviolet light with an inverse zero layer mask. 5) This was followed by post-exposure baking and developing. 6) Finally, after wet oxide etching on the non-features, stripping the photoresist of wafers was done. B) Completed pillar substrate. The size of the square completed pillars is about 3 μm × 3 μm in width and 89 nm in height. C) Root Mean Square (RMS) roughness in a pillar. Atomic force microscopy was used to estimate the roughness of the surface. RMS roughness in a pillar was 0.35nm.

Fig 1A shows the schematic process of the wafer substrate preparation. Prime-grade 300-mm silicon wafers with p-type boron, (1 0 0) orientation, 1 to 5 Ω·cm^-1^, and 725-μm thickness were obtained from Process Specialties. The wafers were deposited with 100 nm thermal oxide by dry oxidation at 1,000°C in a furnace under pure oxygen atmosphere for 2 hours. Commercial photoresist P5107 (Rohm and Haas Chemicals) was spin coated on the wafers at 2,000 rpm for 40 seconds using the RF3S Coat/Develop Track (Sokudo). The wafers were exposed with an inverse zero layer mask using the 248-nm wavelength NSR-S205 KrF Scanner (Nikon). This was followed by post-exposure baking at 110°C for 90 seconds and then developing for 30 seconds using the developer NMD-3 2.38% (TOK America). Wet oxide etching of the wafers was performed using buffered hydrofluoric acid, which was prepared by mixing 5 parts of 40 wt% of ammonium fluoride (Sigma-Aldrich) with 1 part of 49 wt% of hydrofluoric acid (Sigma-Aldrich) for 1 minute. This was followed by stripping the wafers with Nano-Strip (KMG Electronic Chemicals, formerly Cyantek) for 24 hours. Wafers were finally washed with deionized water and sonicated in deionized water for 10 minutes. This results in the completion of substrate preparation in which the feature area has height of 100 nm and contains thermal oxide and the non-feature area contains silicon. The Dimension 5000 AFM system (Veeco) was used to measure the roughness and calculate the density of the substrate. Fig 1A shows the process of silicon wafers preparation, and Fig 1B shows the pillars formed after the process was completed. Fig 1C shows the root mean square roughness of the substrate evaluated by Atomic force microscopy (Digital Instruments, Nanoscope III A). The density of the substrate was calculated to be approximately 100 to 150 pM.

The method used to derivatize the wafer and the details of preparing amino acid activation solution cocktails are described in supporting information (S1 Text).

### Peptide Array Synthesis

**Fig 2:**
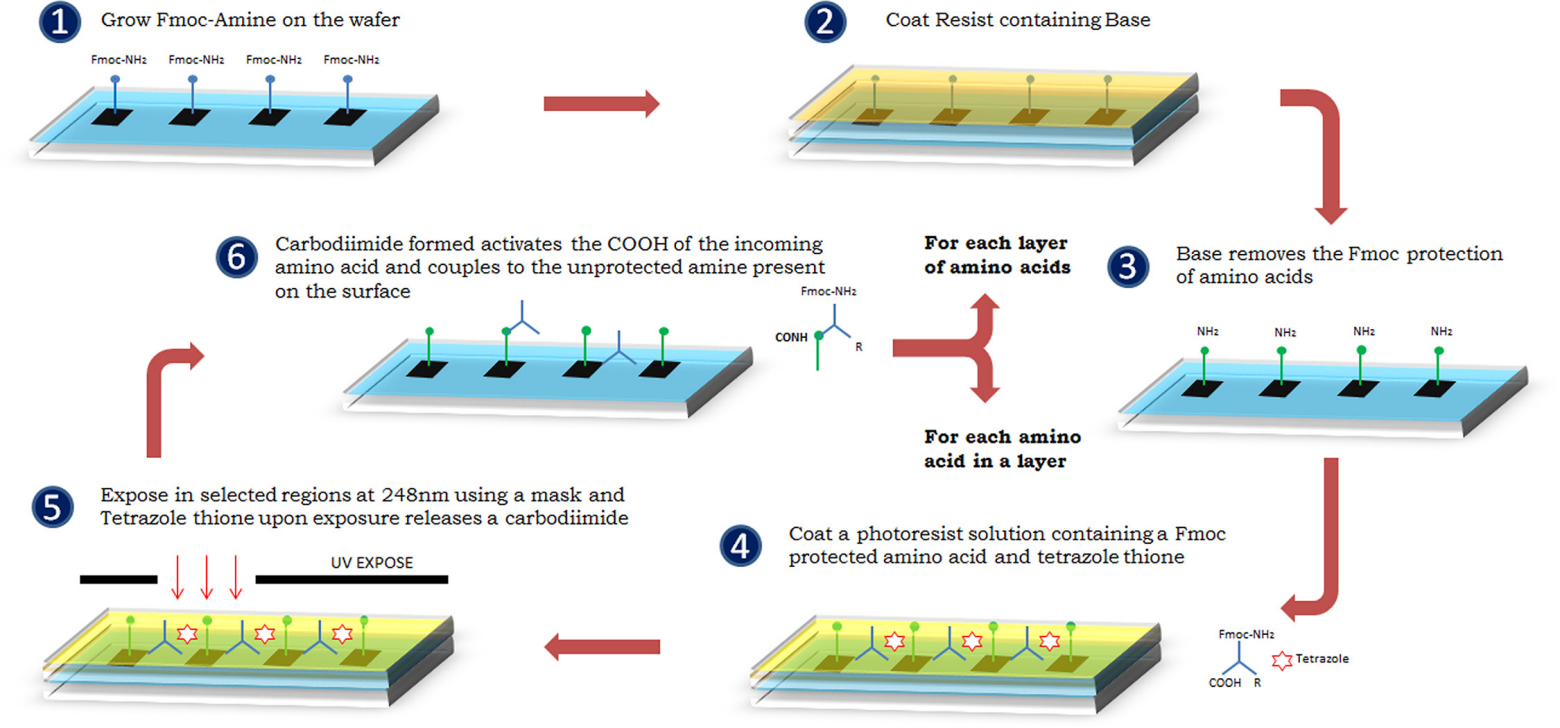
The process for 1-step amine side peptide synthesis. is added 1) Fluorenylmethyloxycarbonyl (Fmoc) amino acids were assembled on the silicon wafer. 2) A base resist solution was coated on the wafer. 3) Fmoc protection was removed in all features, leaving the unprotected amine group. 4) The incoming amino acid activation solution cocktail (includes Fmoc amino acids and tetrazole thione) was added and coated onto a wafer. 5) Then the wafer is exposed to 248 nm wavelength ultraviolet lights using a reticle. On exposure, tetrazole thione releases a carbodiimide. 6) Lastly, the Fmoc amino acid is activated and coupled to the unprotected amine present on the wafer, completing the coupling of 1 layer of amino acid.

The process for 1-step amine side peptide synthesis is shown in Fig 2. A base resist solution containing 1 wt% of polymer and 3 wt% of piperidine dissolved in N-methylpyrrolidone (NMP) is spin coated onto the wafer at 3,000 rpm for 30 seconds and soft baked at 65°C for 1 minute in a hot plate. Then the wafer is baked at 80°C for 300 seconds. Fluorenylmethyloxycarbonyl (Fmoc) protection is removed in all features, leaving the unprotected amine group. The incoming amino acid activation solution cocktail is spin coated onto a wafer at 3,000 rpm for 30 seconds and soft baked at 65°C for 1 minute in a hot plate. Then the wafer is exposed using a reticle which exposes desired features for which the incoming amino acid needs to be coupled at an exposure dose of 120 mJ/cm^2^ and then hard baked at 85°C for 90 seconds in a hot plate. On exposure, tetrazole thione releases a carbodiimide, and selective activation of amino acid is achieved in the exposed features. Therefore, the incoming Fmoc amino acid present in the cocktail is activated and coupled to the unprotected amine present on the wafer in the same step, completing the coupling of 1 layer of amino acid. Each layer of coupling comprises reticles for each incoming Fmoc amino acid to be coupled, which exposes features independent of the rest of the reticles used for the same layer. After the completion of all amino acids for a particular layer, the wafer is then spin coated with a solution of 50 wt% of NMP and 50 wt% of acetic anhydride to cap any unprotected amines remaining in any part of the wafer which have not been coupled with the next amino acid in the layer. The wafer is stripped in acetone and isopropyl alcohol (BDH Chemicals) to remove any resist present on the surface after each step. The whole process is repeated for each individual layer of amino acid designed to be coupled to complete the synthesis of all peptides designed using the reticles. The method used to verify the purity level of the synthesized peptide and the details of fluorescein quality control are described in supporting information (S2 Text).

#### Side Chain Protection Removal

After the completion of peptide synthesis, the side group protection present for some amino acids needs to be removed to enable biological activity of the peptide. Side chain Protection Removal cocktail is prepared by mixing 95 weight % Trifluoroacetic Acid [TFA] (Sigma Aldrich) and 5 weight % DI Water. Wafers are reacted with side chain protection removal cocktail for 90mins. This is followed by washing the chips successively with TFA (5mins), isopropyl alcohol (5mins), NMP (5mins), neutralize with 5 wt% of Ν,Ν-Diisopropylethylamine (Alfa Aesar) in NMP (5mins) followed by washing the wafer successively with NMP (5mins) and isopropyl alcohol (5mins).

### Peptide Array—Gliadin and modified gliadin Synthesis

**Fig 3:**
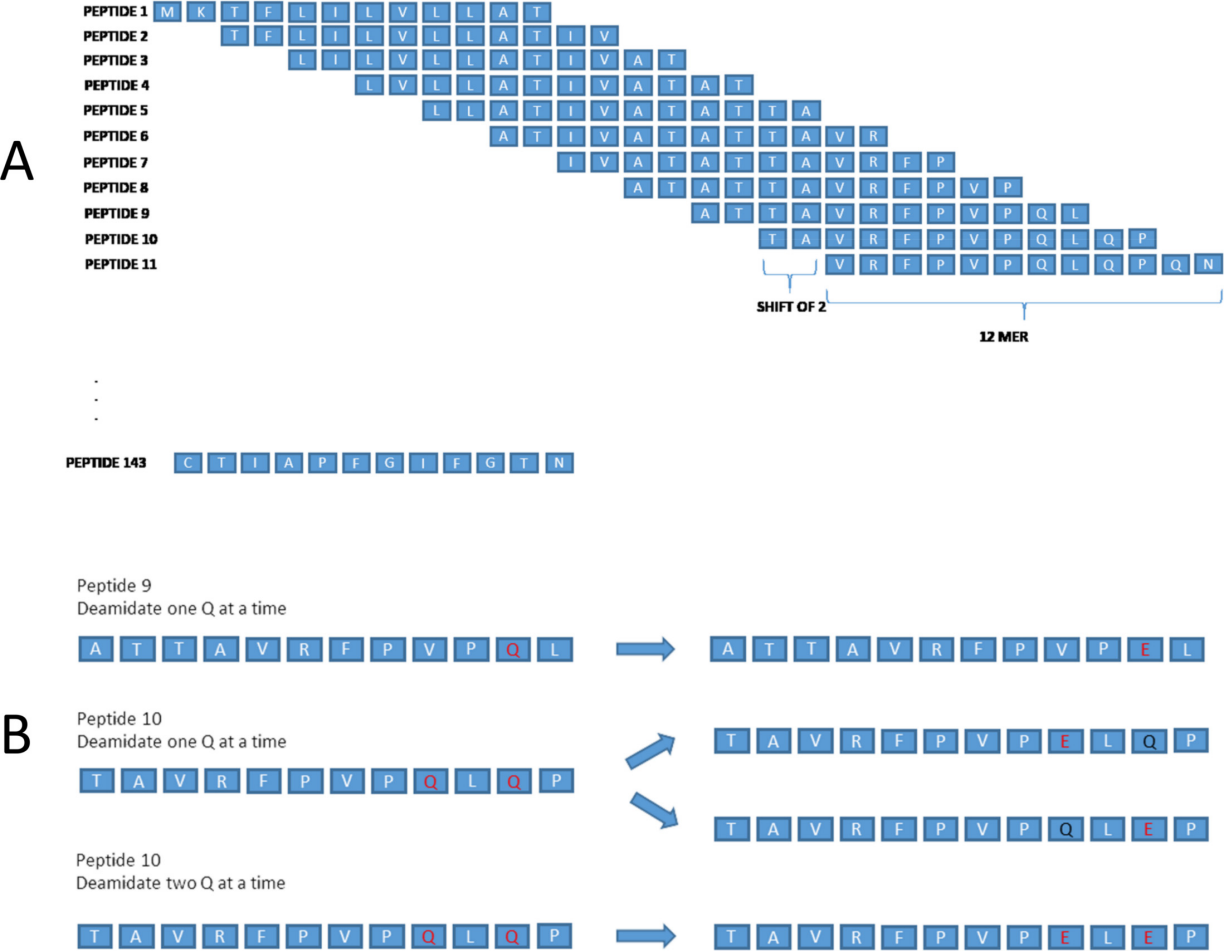
Examples of synthesized overlapping 12-mer peptide sequences of alpha gliadin. A) Examples of overlapping 12 amino acids long peptides with a lateral shift of 2 amino acids covering the alpha gliadin sequence are shown. B) Examples of modification (substitution of glutamic acid for glutamine) of native gliadin sequences 1 at a time and 2 at a time.

We constructed a library of gliadin peptides by building each 12-mer-long peptide with a lateral shift of 2 amino acids of α, β, γ, and Ω fractions of gliadin (Fig 3A). Moreover, we also established a library of modified gliadin peptides with deamidation sites incorporated into the gliadin sequences taken from the aforementioned library of gliadin peptides. In synthesizing the peptides for the library of modified sequences, glutamic acid (E) was inserted in place of glutamine (Q) reflecting the deamidation process and this was done 1, 2, and 3 at a time (Fig 3B). For example, for the original sequence, FLQQPQQPSP, the following sequences were synthesized: FLEQPQQPSP, FLQEPQQPSP, FLEEPQQPSP, FLEEPEQPSP, FLQQPQEPSP and so on (Fig 3B). Original sequences are termed as native gliadin sequences and those sequences in which glutamic acid are incorporated in place of glutamine, are referred to as modified gliadin peptides. These gliadin peptide sequences from the gliadin peptides library were all synthesized using a proprietary combinatorial photolithographic process which was explained in Fig 2 as described above. The amino acid sequences of α, β, γ, and Ω gliadin used for this process are shown in S1 Table.

### Peptide Array Analyzer

To determine the immune-reactive peptide sets, immunologic binding assays with clinically proven celiac positive samples, which were from the exploratory cohort, were performed on an array that included the overlapping peptide sequences of α, β, γ, and Ω gliadin. Using the fluorescence microarray scanner, immunoassay binding activity was scanned. The scanned immunoassay binding data were analyzed to differentiate the substantial levels of binding, which referred to the mean signal binding intensity of the subsequences. After the completion of bioassay, fluorescent binding intensities obtained are converted to antibody-binding units after normalizing the values for each peptide. Peptide values are normalized using a least variant set method of normalization algorithm. Further analysis for determining the sensitivity and specificity of each peptide is done using the receiver operating characteristic (ROC) curve. Threshold value for the ROC curve for each peptide is determined by choosing the value which has the highest sensitivity and specificity.

Overall sensitivity and specificity are calculated as follows. For a given specificity range, a sample is considered to be positive if any one of the peptides belonging in the specificity set has antibody normalized units greater than twice the maximum control sample antibody normalized units, which are calculated based on each specificity set.

## Results

### Algorithm to Determine B-cell Epitopes

**Fig 4:**
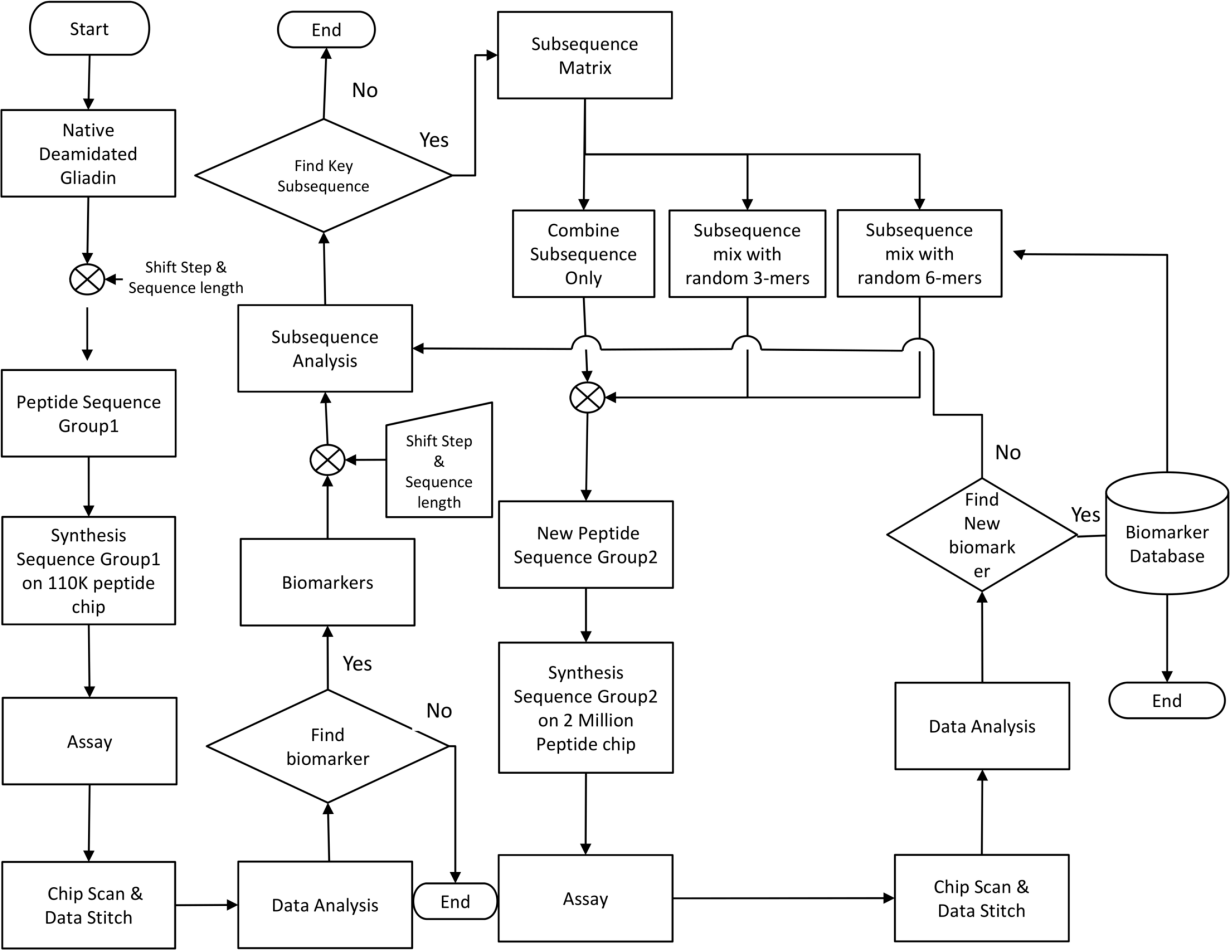
Flowchart for Biomarker Selection. Overlapping 12-mer peptide sequences of α, β, γ, or Ω fractions of gliadin and modified gliadin peptides sequences (substitution of glutamic acid for glutamine, MGPs), were synthesized on a 110,000 array. Deamidation is performed by replacing certain glutamine (Q) present in the sequence with glutamic acid (E). These synthesized peptides sequences were tested for antibody binding to celiac positive samples. After obtaining data of binding intensity from the immunoassay, the most frequently occurring key subsequences were identified. Based on these key subsequences, a new peptide subsequences, which is the combination of key 3-mer subsequences and random 3-and 6-mer subsequences, was formed to in silico form. (Random sequences were generated using a simple random generator program in MATLAB.) These newly assembled sequences were then synthesized on a peptide array to perform the immunoassay with celiac positive samples for identifying the new biomarkers to distinguish CD from controls.

**Fig 5:**
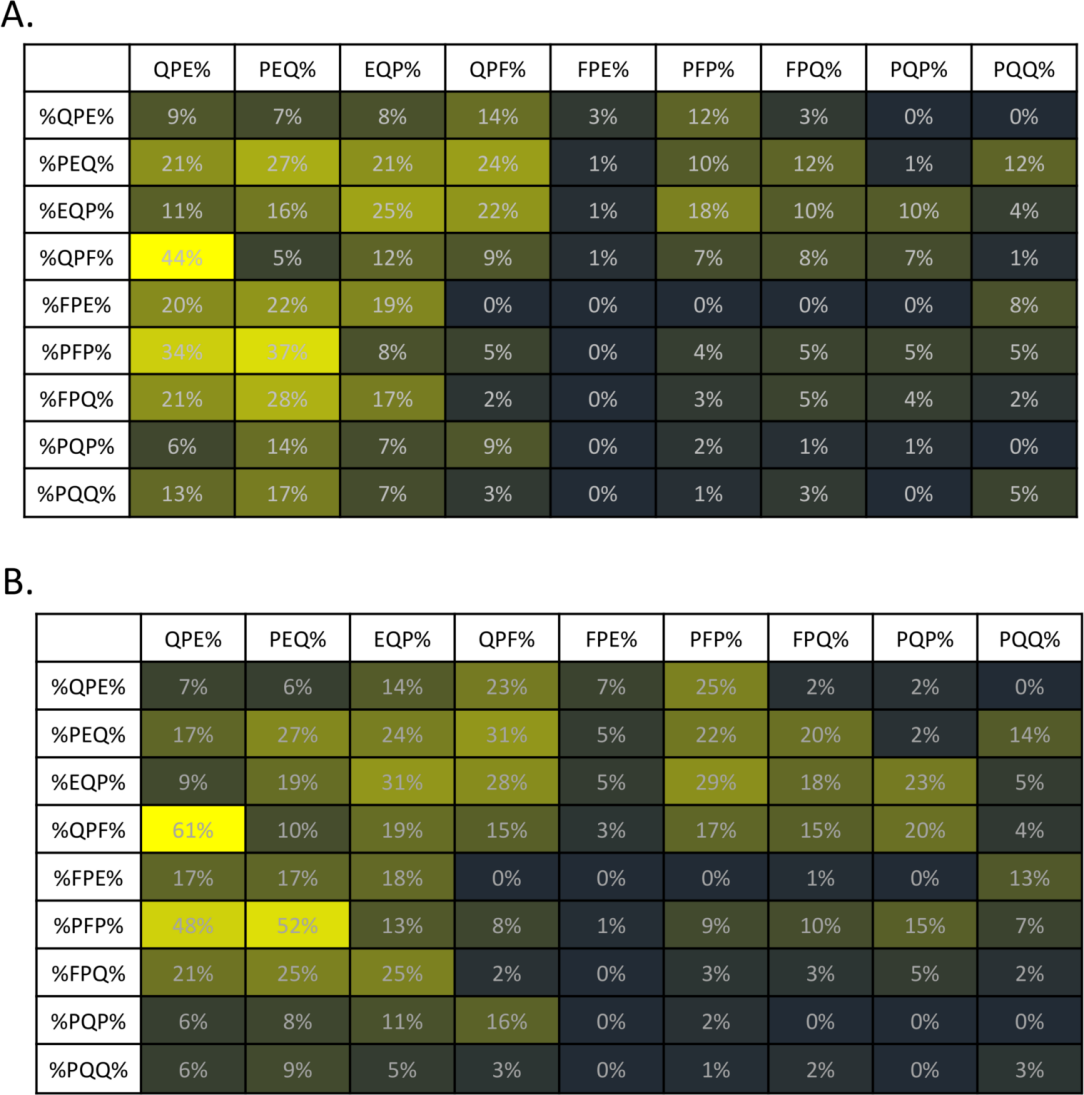
Celiac Subsequence Matrix. The 3-mer subsequences with maximum occurrences among sequences with high sensitivity and specificity for among IgG and IgA reactivity were determined, and the best combinations of subsequences were plotted as a matrix table. The nine 3-mers with the highest score were combined in each possible iteration of two 3-mers to form 81 6-mer sequences, with N-terminal sub-sequences listed in the column, and the C-terminal sub-sequences listed in the row. A) IgG 3-mer subsequences, maximum occurrences. B) IgA 3-mer subsequences, maximum occurrences. % implies any number of amino acid(s) present prior to the subsequence portion of the sequence. For example, ‘%QPE’ could be WALLTYQPE while ‘%QPE%’ could be WAQPELLTY.

Fig 4 shows an algorithm to determine B-cell epitopes from native and modified gliadin-derived peptides (MDPs, substitution of glutamine with glutamic acid). The 110K Ver2.0 (Vibrant Sciences), a peptide microarray consisting of 110,000 overlapping peptides (12-mer sequences) that represent gliadins and MGPs in duplicates (Fig 3A), was synthesized and picked and placed onto 96 pillar plates. Using this peptide microarray, 188 serum samples from a set consisting of 90 CD patients and 98 controls were tested for IgG and IgA reactivity to synthesized gliadin peptides to determine the key set of sequences (3-mer peptides) with the highest occurrences among celiac samples (Fig 5). First, each 12-mer sequence on the peptide microarray was assessed for binding by sera from celiac positive individuals. Within the set of all 12-mer sequences that showed weakly or strongly positive binding signals to sera from celiac patients, the most frequently occurring subsequences (3-mer) were determined using a computer program. Fig 5 shows the nine most frequently occurring 3-mer sequences, relative to IgA (Fig 5A) or IgG (Fig 5B) isotype. A matrix table (Fig 5) shows the percentage of occurrence of these sequences in a specific order. After identifying the most informative, frequent 3-mer subsequences, these 3-mer peptides were paired into 6-mers in forward and reverse combinations and then in situ synthesized into the second phase of arrays. Forward combination refers to the peptides with informative subsequence at the beginning of the sequence preceding a random subsequence. Reverse combination refers to sequences designed by having the random portion at the beginning followed by the informative subsequence. For example: **QPFQPE**AGLTHG is forward combination sequence whereas AGLTHG**QPFQPE** is a reverse combination sequence-thick letters designate the informative sequences. These highly reactive sequences could then be synthesized with learning from the first library; this set is continually evolving as more samples are run. Interestingly, reversing the sequences substantially impairs the recognition (S2 Table). This suggests that antibody recognition of epitopes of DGPs in CD is highly sequence-specific and only a few sequences are recognized across the entire spectrum of patients with CD. However, we cannot exclude the possibility that low reactivity in reversed sequences may be related to sterical hindrance imposed by peptide anchoring to the chip. Finally, 2 distinct consensus gliadin peptide sets (#1 and #2, S3 Table) were identified for discriminating patients with CD from controls in the cohort; peptide set 1 (IgG) exhibited 80% (95% CI, 71–87) sensitivity and 85% (95% CI, 76–91) specificity, and peptide set 2 (IgA) exhibited 86% (95% CI, 77–91) sensitivity and 89% (95% CI, 82–94) specificity. Table 2 shows the detailed discriminant power of these identified peptide sets for the diagnosis of CD in the exploratory cohort.

**Table 2:** Discriminant Power of Identified Peptide Sets (Continuous Gliadin Sequences) for the Diagnosis of CD in the Training Cohort.

Peptide Set	Celiac disease* N = 90	Controls N = 98	Total (n)
Peptide Set #1 (IgG)			
Positive	72	15	87
Negative	18	83	101
Peptide Set #2 (IgA)			
Positive	77	11	88
Negative	13	87	100

*Celiac Disease was defined by the current standard diagnosis of CD, composed of CD serology and duodenal biopsy.

### Determination of Novel Discontinuous B-cell Epitopes

**Fig 6:**
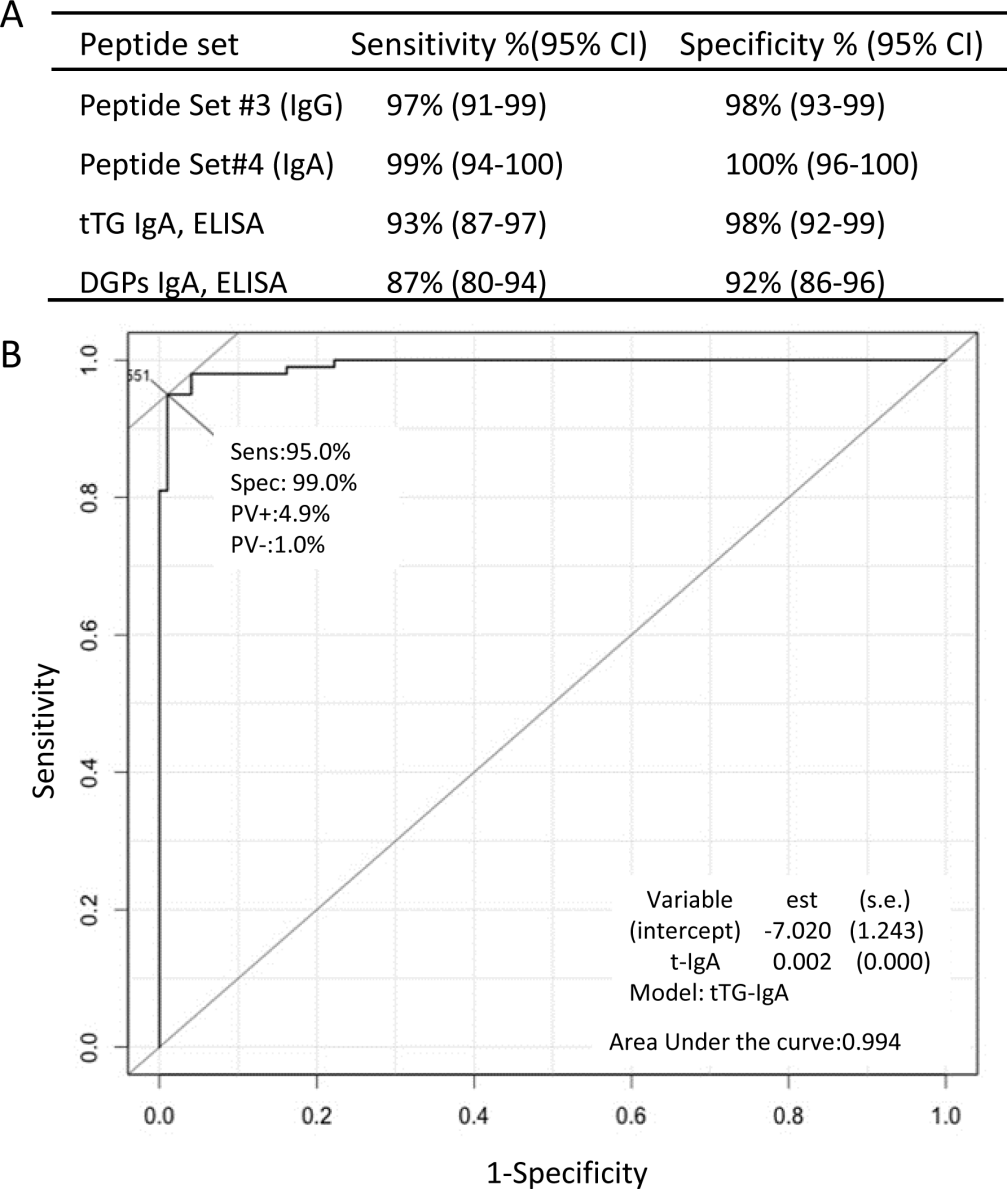
Sensitivity and Specificity of the Novel Peptide Sets for CD (A) and ROC Curve (B). A) Discriminant power of the peptide sets (#3 and #4) of discontinuous B-cell epitopes for the diagnosis of CD, compared to the currently used ELISA kits for the diagnosis of CD (Inova Diagnostics). These kits measure IgA antibodies to tTg and IgA antibodies to DGPs. B) Example of a synthetic DGP with a high area under the curve (0.99). The ROC curve is plotted on the basis of 1-specificity and sensitivity under each threshold for each sequence. CD indicates celiac disease; modified gliadin peptides, which glutamic acid was substituted for glutamine; ELISA, enzyme-linked immunosorbent assay; ROC, receiver operating characteristic; tTg, tissue transglutaminase. The amino acid sequences of peptide set #3 and #4 are shown in S2 Table.

To improve diagnostic accuracy for CD, we assembled a novel set of sequences by combining sequences with identified high frequency of 3-mer sequences in the first step and random 3-mer and 6-mer peptides to form peptides between 6 and 15 amino acids in length (Fig 4). Examples of discontinuous peptide sequences, which were combination of the frequent 3-mer sequences from DGP and randomly assigned peptides, are presented in S3 Fig. With the ROC curves for discriminating patients with CD from controls, which were constructed for each synthesized peptide, high sensitive and highly specific peptide sets were identified. To evaluate the accuracies of these selected peptide biomarkers for the diagnosis of CD, we used Random Forest,[17] a statistical algorithm that creates voting classes of decision-making trees to evaluate the significance of each marker and classify samples. We identified 2 distinct discontinuous gliadin sequence sets that, when combined, significantly improved the sensitivity (IgG, 97%; IgA, 99%) and specificity (IgG, 98%; IgA, 100%) (*P* < .001) for the diagnosis of CD, in contrast to the first sets of peptides of gliadin sequences or current standard CD serologic testing with enzyme-linked immunosorbent assay kits, and the sensitivity and specificity of these current standard CD serologic testing shown in Fig 6A are similar to other previous studies.[18] These 2 distinct peptide sets are shown in S3 Table. The cross-validated area under the curve of a ROC curve using modified sequences, which glutamic acid was substituted for glutamine, for predicting CD was 0.99 (Fig 6B). Synthesis of these two distinct peptide sets of sequences was done on a silicon-based peptide array platform to confirm the discriminative power in another CD cohort.

### Confirmation of the Novel B-cell Epitopes

**Fig 7:**
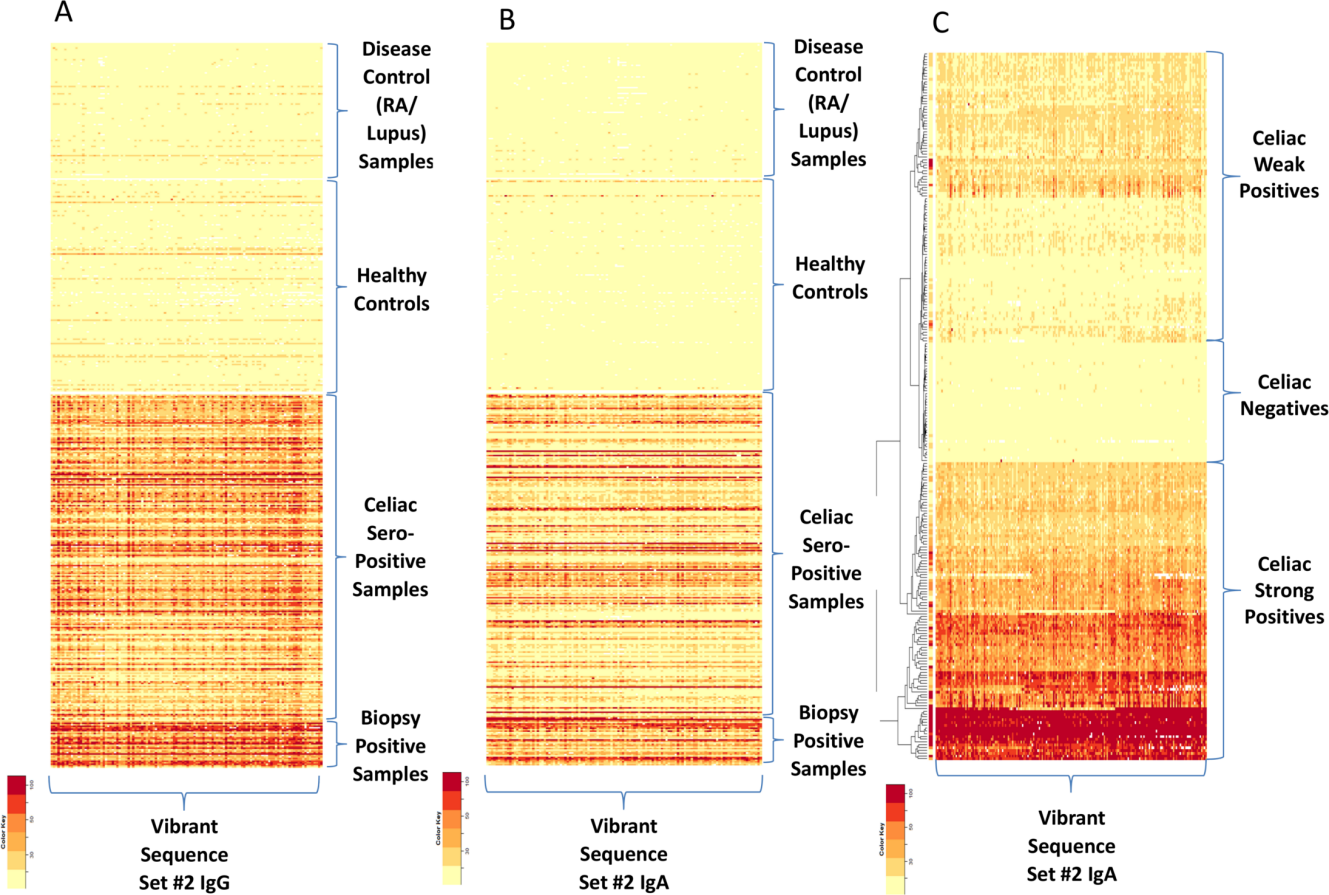
Heat Maps of Antibody-Binding Intensity in Validation Set Samples. A) Heat-map shows antibody-binding data for a peptide set #3 (IgG) with high significance values to differentiate samples seropositive for celiac disease (CD) from controls and disease controls. B) Heat map shows antibody-binding data for a peptide set #4 of IgA. C) Heat map shows the natural subgrouping of CD weak positives and strong positives and negatives based on a hierarchical clustering algorithm[27] from a peptide analyzer in a confirmatory cohort. RA indicates rheumatoid arthritis.

**Fig 8:**
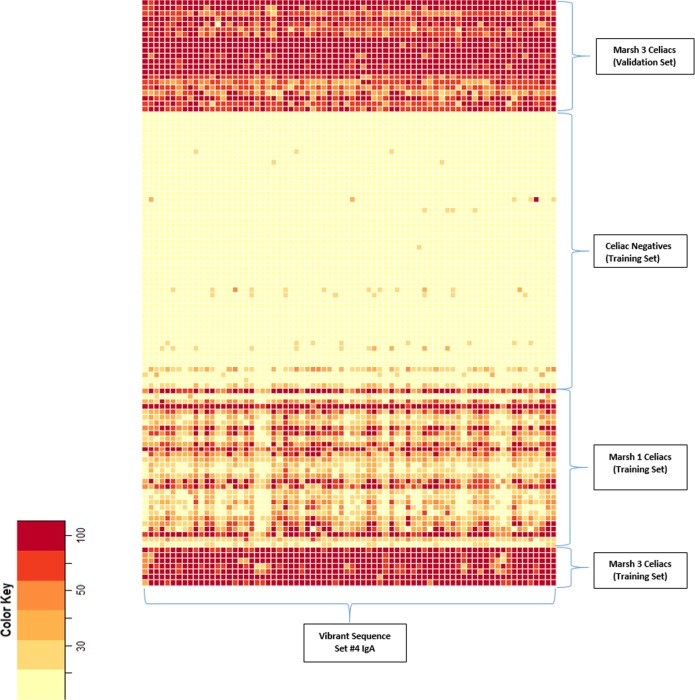
Heat Map Based on Duodenal Pathology With Marsh Classification. The heat map shows 2 clusters of high or low antibody-binding intensity of the identified peptide in the set. Thirty-three patients with celiac disease autoimmunity in whom the disease was subsequently diagnosed after blood was drawn in the confirmatory cohort also showed high binding intensity, which was similar to the high-intensity group in the exploratory set.

To confirm the discriminative power of the novel discontinuous peptides from the exploratory cohort, we assayed sera from a population cohort of 1,896 persons in a blinded test. This cohort comprised 306 persons who were seropositive to CD and 1,590 controls. “Seropositive to CD” was defined as positivity for both anti–tissue transglutaminase IgA and anti-endomysial antibodies, which together have a high predictive value for biopsy-proven CD[19, 20]; assays of these antibodies are the current standard serologic tests for CD. Two novel synthetic peptides sets comprising discontinuous gliadin sequences showed a high accuracy for distinguishing CD cases from controls, achieving 100% agreement for the positive CD cases (Fig 7A and 7B). More interestingly, CD cases could be separated into a lower- or a higher-reactivity group on the basis of antibody-binding intensity (Fig 7C); the sera of all 33 patients who subsequently had clinically diagnosed CD exhibited higher-intensity binding and belonged to a higher-reactivity group. Further, to evaluate the correlation between duodenal pathologic findings of CD and immune reactivity to the identified peptide sets, we used the sera of 48 biopsy-proven CD cases and 33 CD cases that were subsequently diagnosed by duodenal pathology during follow-up (of the 306 seropositive CD cases). None of the first synthesized peptide sets from gliadin sequences could categorize serum samples based on the Marsh Score for small-intestinal pathology; however, newly assembled peptide sets with discontinuous gliadin sequences could clearly distinguish between less severe duodenal lesions and Marsh stage 3 (Fig 8).

## Discussion

In CD, some B-cell epitopes of gliadin are linear.[21, 22] It is well known that antibodies primarily recognize 3-dimensional structures as opposed to linear structures, such as peptides presented by MHC molecules on antigen presenting cells to T cells. Knowing as we do that the immune response typifying CD requires proteins from wheat and like grains, we have been able to use the discovery power of this novel ultra-throughput platform to systematically search linear continuous and discontinuous peptides derived from all of the proteins known to be immunogenic for CD. Here, using this powerful new method, we identified a set of novel epitopes, which are recognized by the circulating antibodies found in the sera of patients with CD. These novel peptides are derived from modified gliadin (substitution of glutamic acid for glutamine) and show high sensitivity and specificity for discriminating patients with CD from controls. These 9-mer to 15-mer sequences, which are different from the known 33-mer gliadin sequence,[23] represent novel and heretofore unidentified B-cell epitopes of gliadin. The identified peptides were subjected to a rigorous test of significance and predictive values by testing serum samples from a confirmatory community-based cohort. We found that the identified novel epitopes from gliadin have a high accuracy in distinguishing patients with CD from controls, showing 99% sensitivity and 100% specificity.

The biomarker discovery via the platform of highly efficient mass manufacturing of ultra-high-density peptide microarrays presented here provides an efficient method to determine novel epitopes through mapping of antigens and combining the immunopotent sequences with random peptides. Indeed, the silicon-based platform allows several million peptides to be synthesized in parallel and makes it possible to apply the known antigens with overlapping peptides on a single microarray. We have used peptide arrays based on 2.1 million 9-mer to 15-mer peptides, each overlapping with 3 or 6 amino acids, to cover the immunogenic proteins with very high density, maximizing our ability to identify informative peptides, and we showed the effectiveness and utility of this technology in identification of unknown but novel epitopes that are recognized by patients with autoimmune disease. This advance will allow for the development of more precise diagnostic tests that can be incorporated into panels of testing for autoimmune diseases, including CD. Moreover, we have also evaluated the contribution of the individual amino acids of the antigen for antibody binding by designing microarrays of peptides containing lateral shifts of 1 amino acid, achieving higher mapping resolution for the target antigen.

All previous photolithography-based microarray in situ synthesis methods [5, 24–26] are based on individually addressable de-protection steps and then coupling of monomers to those selective de-protected sites. Despite advances in peptide microarray technology, specifically using photolithography on silicon chips to selectively de-protect amino acids that follow generalized activation, peptide microarrays are still challenging because of low yields, less fidelity, and time-consuming production methods. In contrast, the novel method described here involves generalized de-protection with selective activation; similar to the protein synthesis process that occurs in nature, in which generalized de-protection is followed by selective activation. This provides 2 major advantages: 1) a far-higher fidelity of peptide synthesis, and 2) a greatly reduced time requirement for each step. This can also permit a much higher number of steps, as many as 400, in the synthesis of the peptide array, resulting in a very low loss of yield. Prior methods, which work on selective de-protection followed by generalized activation, have a lower fidelity over time and lead to much higher loss of yield and less accuracy in the production of peptides. It is this combination of high fidelity and much shorter production time that results in a much higher yield and the ability to generate a large number of chips quite inexpensively with the very high fidelity required for biomarker discovery and ultimately for high-throughput diagnostic testing. Moreover, we developed the software algorithms with an evolutionary computational approach that is used to predict and then manufacture the appropriate matrices of combinations of peptide sequences. This highly efficient multi-step process combines off-the-shelf software (described in the Materials and Methods section) and a proprietary algorithm. Our method uses the state-of-the-art 248-nm semiconductor lithography tools on a proven 300-mm silicon wafer platform. Our very high microarray density enables not only the molecular diversity needed for biomarker discovery but also large-scale biomarker validation. In addition, this method is well suited for mass manufacturing for routine diagnostics, since the chip size can scale down to 0.5X 0.5 mm^2^ to fit any diagnostics well plate format (eg, 96, 384, 1396). This also enables smaller samples to be used for routine diagnostics.

In summary, because the prevalence of CD is increasing, the development of high-fidelity diagnostic tests with a high degree of accuracy that can be undertaken on a large scale will be an important addition to the diagnostic armamentarium for CD and potentially other immune diseases. Such a test will be important not only for clinical care but also for understanding the pathogenesis of disease. The relative noninvasiveness, broad availability, and versatility of the high-throughput peptide microarrays make this technology well suited for incorporation into routine health care and also provide a promising new tool for biomarker discovery.

## Supporting Information

S1 Fig Analysis of Peptide Purity. Peptide LKWLDSFTEQ was synthesized and cleaved from the wafer substrate. Mass spectrometry shows the synthesized peptide mass, which is matched to the expected mass.
(DOCX)

S2 Fig Fluorescein Quality Control. From the heat map data, it is determined that the important amino acids in this sequence are L**K**W**LDS**FTEQ with the key amino acids highlighted using red color. If any other amino acid is used in place of the key amino acid, the sequence does not display any biological activity.
(DOCX)

S3 Fig Examples of discontinuous peptides subsequence. Thick red letters indicate the amino acid linkers from the frequent 3-mer subsequences of modified gliadin peptides (substitution of glutamic acid for glutamine), which are identified by the immunoassays with celiac samples, while other letters in each sequence indicate the randomly assigned amino acids.
(DOCX)

S1 Table Alpha, Beta, Gamma and Omega Gliadin peptides sequences. (DOCX)

S2 Table Examples for two sequences grown in the original and reverse direction to check the binding intensity with celiac patients’ samples. (DOCX)

S3 Table 12-mers amino acid subsequences of four distinct peptide sets (#1, #2, #3 and #4). (DOCX)

S1 Text Derivatization and Amino Acid Activation Solution. (DOCX)

S2 Text Peptide Purity and quality control. (DOCX)
