# Supplementary material for: Determination of B-Cell Epitopes in Patients with Celiac Disease: Peptide Microarrays
Source: PLoS One. 2016 Jan 29;11(1):e0147777. doi: 10.1371/journal.pone.0147777 (PMC4732949; doi:10.1371/journal.pone.0147777)
Supplement: S1 Fig — Peptide LKWLDSFTEQ was synthesized and cleaved from the wafer substrate. Mass spectrometry shows the synthesized peptide mass, which is matched to the expected mass. (DOCX) [file pone.0147777.s001.docx]

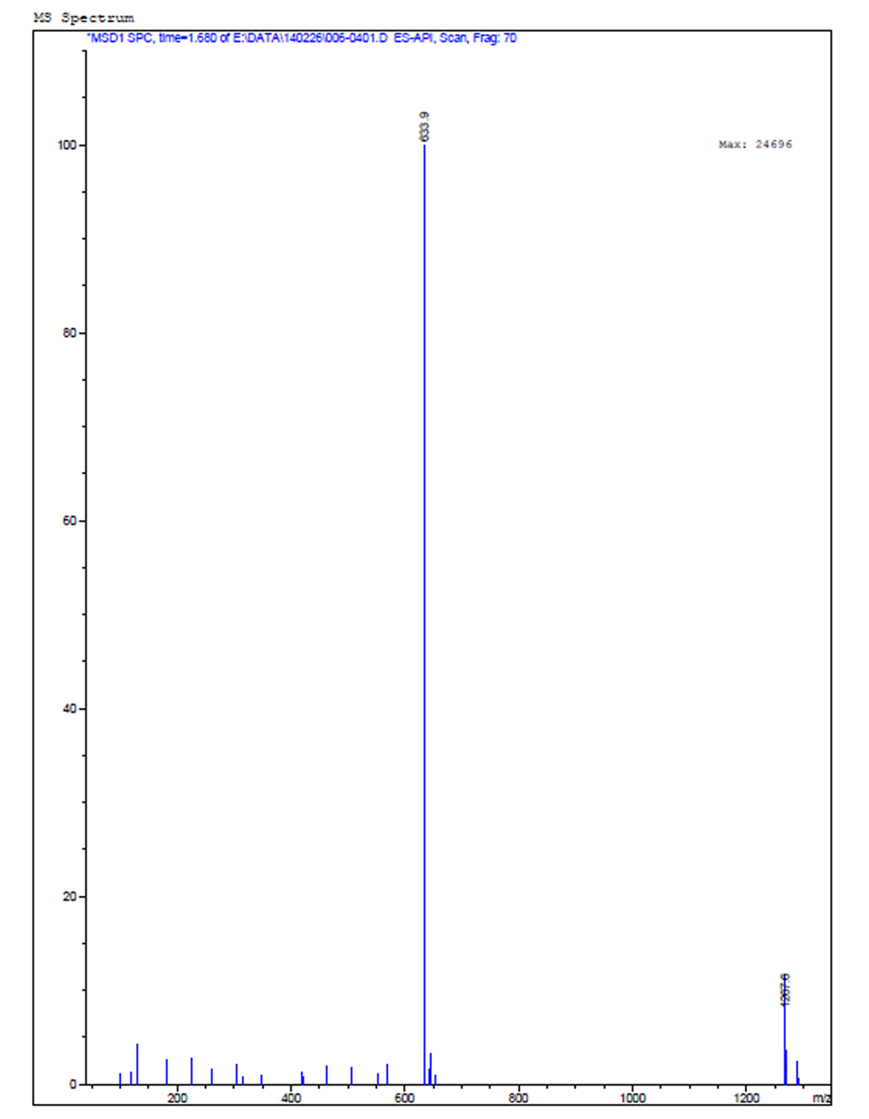


**S1 Figure. Analysis of Peptide Purity.** Peptide LKWLDSFTEQ was synthesized and cleaved from the wafer substrate. Mass spectrometry shows the synthesized peptide mass, which is matched to the expected mass.
