# Supplementary material for: Determination of B-Cell Epitopes in Patients with Celiac Disease: Peptide Microarrays
Source: PLoS One. 2016 Jan 29;11(1):e0147777. doi: 10.1371/journal.pone.0147777 (PMC4732949; doi:10.1371/journal.pone.0147777)
Supplement: S2 Fig — From the heat map data, it is determined that the important amino acids in this sequence are LKWLDSFTEQ with the key amino acids highlighted using red color. If any other amino acid is used in place of the key amino acid, the sequence does not display any biological activity. (DOCX) [file pone.0147777.s002.docx]

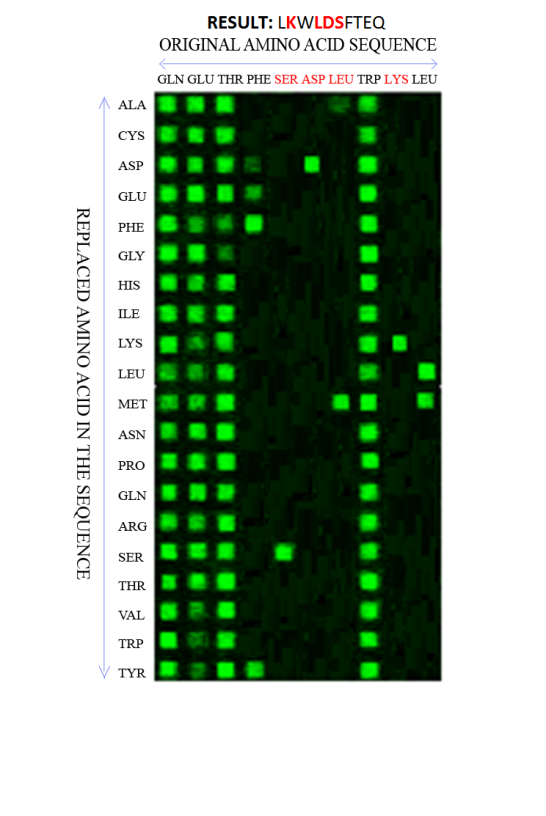


**S2 Figure. Fluorescein Quality Control.** From the heat map data, it is determined that the important amino acids in this sequence are L**K**W**LDS**FTEQ with the key amino acids highlighted using red color. If any other amino acid is used in place of the key amino acid, the sequence does not display any biological activity.
