# Supplementary material for: Determination of B-Cell Epitopes in Patients with Celiac Disease: Peptide Microarrays
Source: PLoS One. 2016 Jan 29;11(1):e0147777. doi: 10.1371/journal.pone.0147777 (PMC4732949; doi:10.1371/journal.pone.0147777)
Supplement: S3 Fig — Thick red letters indicate the amino acid linkers from the frequent 3-mer subsequences of modified gliadin peptides (substitution of glutamic acid for glutamine), which are identified by the immunoassays with celiac samples, while other letters in each sequence indicate the randomly assigned amino acids. (DOCX) [file pone.0147777.s003.docx]

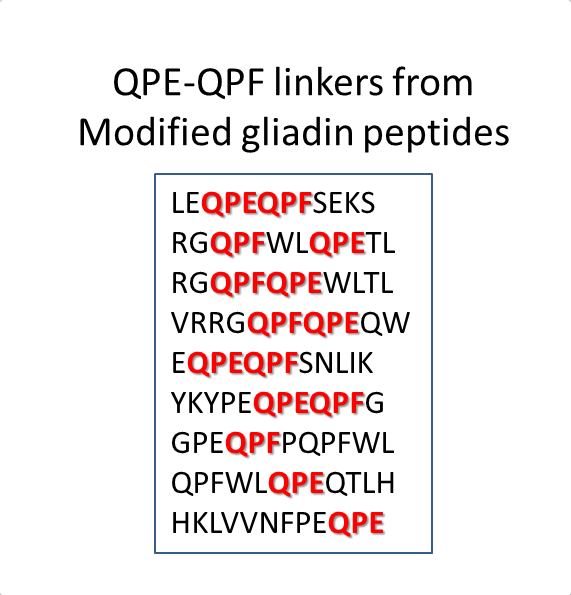


**S3 Figure.** **Examples of discontinuous peptides subsequence.** Thick red letters indicate the amino acid linkers from the frequent 3-mer subsequences of deamidated gliadin peptides, which are identified by the immunoassays with celiac samples, while other letters in each sequence indicate the randomly assigned amino acids.
