# Supplementary material for: Determination of B-Cell Epitopes in Patients with Celiac Disease: Peptide Microarrays
Source: PLoS One. 2016 Jan 29;11(1):e0147777. doi: 10.1371/journal.pone.0147777 (PMC4732949; doi:10.1371/journal.pone.0147777)
Supplement: S1 Table — (DOCX) [file pone.0147777.s004.docx]

**S1 Table. Alpha, Beta, Gamma and Omega Gliadin peptides sequences**

| Gliadins | Amino acid sequences |
| --- | --- |
| Alpha Gliadin sequence | MKTFLILALL AIVATTATTA VRVPVPQLQP QNPSQQQPQE QVPLVQQQQF LGQQQPFPPQ QPYPQPQPFP SQQPYLQLQP FPQPQLPYSQ PQPFRPQQPY PQPQPQYSQP QQPISQQQQQ QQQQQQQQQQ QQQQQILQQI LQQQLIPCMD VVLQQHNIAH GRSQVLQQST YQLLQELCCQ HLWQIPEQSQ CQAIHKVVHA IILHQQQKQQ QQPSSQVSFQ QPLQQYPLGQ GSFRPSQQNP QAQGSVQPQQ LPQFEEIRNL ALQTLPAMCN VYIPPYCTIT PFGIFGTN |
| Beta Gliadin Sequence | MKTFLILVLL AIVATTATTA VRFPVPQLQP QNPSQQQPQE QVPLVQQQQF LGQQQPFPPQ QPYPQPQPFP SQLPYLQLQP FPQPQLPYSQ PQPFRPQQPY PQPQPQYSQP QQPISQQQQQ QQQQQQQQQQ QQQILQQILQ QQLIPCMDVV LQQHNIAHGR SQVLQQSTYQ LLQELCCQHL WQIPEQSQCQ AIHNVVHAII LHQQQKQQQQ PSSQVSFQQP LQQYPLGQGS FRPSQQNPQA QGSVQPQQLP QFEEIRNLAL QTLPAMCNVY IPPYCTIAPF GIFGTN |
| Gamma Gliadin Sequence | PQQPFPLQPQ QSFLWQSQQP FLQQPQQPSP QPQQVVQIIS PATPTTIPSA GKPTSAPFPQ QQQQHQQLAQ QQIPVVQPSI LQQLNPCKVF LQQQCSPVAM PQRLARSQML QQSSCHVMQQ QCCQQLPQIP QQSRYQAIRA IIYSIILQEQ QQVQGSIQSQ QQQPQQLGQC VSQPQQQSQQ QLGQQPQQQQ LAQGTFLQPH QIAQLEVMTS IALRILPTMC SVNVPLYRTT TSVPFGVGTG VGAY |
| Omega Gliadin Sequence | ARQLNPSDQE LQSPQQLYPQ QPYPQQPY |
