# Supplementary material for: Determination of B-Cell Epitopes in Patients with Celiac Disease: Peptide Microarrays
Source: PLoS One. 2016 Jan 29;11(1):e0147777. doi: 10.1371/journal.pone.0147777 (PMC4732949; doi:10.1371/journal.pone.0147777)
Supplement: S2 Table — (DOCX) [file pone.0147777.s005.docx]

**S2 Table.** Examples for two sequences grown in the original and reverse direction to check the binding intensity with celiac patients samples.

|  | **Antibody Binding Units** | |
| --- | --- | --- |
| **RRGQPFWQPELT** | **Original Sequence**  **(**RRGQPFWQPELP) | **Reversed Sequence**  **(**PLEPQWFPQGRR) |
| Celiac Disease Sample 1 | 56.41 | 1.65 |
| Celiac Disease Sample 2 | 78.99 | 2.14 |
| Celiac Disease Sample 3 | 18.63 | 7.89 |
| Celiac Disease Sample 4 | 6.51 | 4.18 |
| Celiac Disease Sample 5 | 59.74 | 9.63 |

|  | **Antibody Binding Units** | |
| --- | --- | --- |
| **NQPEQPFPLPVA** | **Original Sequence**  **(**NQPEQPFPLPVA) | **Reversed Sequence**  **(**AVPLPFPQEPQN) |
| Celiac Disease Sample 1 | 88.47 | 8.66 |
| Celiac Disease Sample 2 | 75.33 | 1.56 |
| Celiac Disease Sample 3 | 10.68 | 1.45 |
| Celiac Disease Sample 4 | 25.32 | 3.98 |
| Celiac Disease Sample 5 | 14.55 | 6.74 |
