# Supplementary material for: Determination of B-Cell Epitopes in Patients with Celiac Disease: Peptide Microarrays
Source: PLoS One. 2016 Jan 29;11(1):e0147777. doi: 10.1371/journal.pone.0147777 (PMC4732949; doi:10.1371/journal.pone.0147777)
Supplement: S3 Table — (DOCX) [file pone.0147777.s006.docx]

**S3 Table. 12-mers amino acid subsequences of two distinct peptide sets (#1, #2, #3 and #4)**

| New peptide sets | Amino acid sequences |
| --- | --- |
| **Peptide Set #1** | QQPRQPFPEQPQ, YSPYQPEQPFPQ, EQPEQPFPQPEQ, IIPQQPEQPFPL, QPEQPFPEQPQ, PEQPEQPFPETE, FPQPQPEQPFPQ, FPQPEQPFPLQG, QTEQPQQPFPEQ, EQPEQPFPEQPE, QPEQPFPEQPQP, LQPEQPFPEQPE, PTPIQPEQPFPE, TIPQQPEQPFPL, QQFIQPEQPFPQ, LEPEQPFPEQPE, QQFPQPEQPFPQ, SFSQQPEQPFPL, PLQPEQPFPEQP, GQPEQPYPEQPF, SYPVQPEQPFPQ, QLFPLPEQPFPQ, EQPEQPFPQTEQ, FLQPQQPFPEQP, QPQQPEQSFPEQ, PETEQPEQPFPE, QQPFQPEQPFPQ, NPLQPEQPFPLQ, QPYPEQPFPQQG, QPFPQPEQPFPL, FPQEQPEQPFPQ, LQPEQPFPEQPQ, IISQQPEQPFPL, PIPQQPEQPFPL, QPFQPEQPFPWQ, TEQPQQPFPEQP, QSIPQPEQPFPQ |
| **Peptide Set #2** | EPQQPEQSFPEQ, PLQPEQPFPEQL, YSPYQPEQPFPQ, EQPEQPFPQPEQ, QPFPQPEQTFPQ, IIPQQPEQPFPL, FLQPEQPFPEQP, EQPEQPYPEQ, QPEQPFPEQPQ, EQPEQPFPQPQQ, PFPEQPEQPFPE, PNPLQPEQPFPL, EPEQPEQSFPEE, PFLEPEQPFPEQ, PEQPEQPFPETE, FPQPQPEQPFPQ, FPQPEQPFPLQG, QLQRPEQQFPQQ, QPEQPEQSFPEQ, PYPQQPEQPFPQ, EQPEQPFPEQPE, QPEQPFPEQPQP, QEPEQPYPQ, PEQPEQPYPEQP, QPQEPEQSFPEQ, LQPEQPFPEQPE, EQPFPEQPEQPF, PTPIQPEQPFPE, TIPQQPEQPFPL, PFPLQPEQPFPQ, QQFIQPEQPFPQ, LEPEQPFPEQPE, QQFPQPEQPFPQ, QQQPFPEQPFPQ, QPFQPEQPFQEQ, SFSQQPEQPFPL, FPQQPEQPYPQQ, QPFPLQPEQPFP, QPFRPEQPYPQP, EQPEQPYPEQEQ, GQPEQPYPEQPF, SYPVQPEQPFPQ, QPEQPEQSFPEE, QLFPLPEQPFPQ, PFPWPQPEQPFP, FPWFPQPEQPFP, QTPIQPEQPFPQ, QPQQPEQSFPEQ, WQPFPQPEQPFP, EQPFLQPEQPFP, PETEQPEQPFPE, QQPEQQYPSVLG, QQPFQPEQPFPQ, PAPIQPEQPFPQ, QPQQPEQSFPEE, PQPQQPEQPFPQ, QPYPEQPFPQQG, PFPEQPEQPYPQ, SQQPEQPFPLQG, FPQEQPEQPFPQ, QPEQPEQSFPQE, RQPFPQPEQPFP, LQPEQPFPEQPQ, EPEQPEQSFPEQ, PFPQPEQPFP, QQLFPQPEQPFP, QQPFPQPEQPFP, IISQQPEQPFPL, EPQQPEQSFPEE, QPFPQPEQPFQE, QQPFPQPEQQFP, PLQPEQPFPPEQ, PLQPEQPFP, QPFQPEQPFPWQ, QSIPQPEQPFPQ |
| **Peptide Set #3** | RRGQPFWQPELT, VVDPEQPQQDCT, GQPFQPEQPWLT, GQPFWLTQPEQP, TATVVDPEQPQQ, YPEQPEQPGSSE, RANHLNQPEQPF, QPFWQPEQPFLT, LHFPEQPEGRNY, NQPEQPFPLPVA, RGQPFQPEQPFW, TRPDLEQPFPQP, HFPEQPEGRNYE, VVRRGQPFWQPE, GQPFWLQPEQPT, RGQPFWQPELTL, RGQPFWLTLQPE, RGQPFWLQPETL, FPEQPEGRNYEA, LVVNFPEQPESD, EQPEQPFSNLIK, VRRGQPFQPEWL, GQPFWLTQPEQL, RRGQPFWLQPET, FPEQPEDGILDI, VRRGQPFQPEQP, GQPFWLQPEQPF, GQPFWLTLQPEQ, ENPEQPEQPFIK, RGQPFWQPEQLT, HKLVVNFPEQPE, RGQPFWLTQPEQ, TQPEQPFVEIPD, MNMQPEQPFGSD, TYKYPEQPEQPG, WNFGQFPEQPED, GQPFWLQPETLH, LTLHFPEQPEGR, NFPEQPESDKLK, VNFPEQPESDKL, TLHFPEQPEGRN, LYLENPEQPEQP, AVEEQPEQPGDW, QFPEQPEDGILD, QPFWLQPEQPTL, FPEQPESDKLKA, GQPFQPEQPFWL, KARFPQPEQLRD, PEQPEQPIKIRI, ALDPTPQPEQPF, LVVRRGQPFQPE, FAAVAQPEQPFC, GQPFWLQPEQTL, YVLTPEQPFPQQ, KARFPQPEQPFL, QPFWLTLHFQPE, EQPFPQPFWLTL, RRGQPFWQPEQP, RGQPFQPEWLTL, QEQPEQPAGTKA, SQPEQPFGMVNC, VRRGPEQPFPQP, GQPFWQPELTLH, LEQPEQPFSEKS, VRRGQPFWLQPE, QPFQPEQPWLTL, FGQFPEQPEDGI, VRRGQPFWQPEL, RDLYLEQPEQPF, QPFQPEQWLTLH, NPEQPEQPIKIR |
| **Peptide Set #4** | LEQPEQPFSEKS, RGQPFWLQPETL, RGQPFQPEWLTL, VRRGQPFQPEQW, EQPEQPFSNLIK, YKYPEQPEQPFG, GPEQPFPQPFWL, QPFWLQPEQTLH, TATVVDPEQPQQ, HKLVVNFPEQPE, VVDWIQPEQPQQ, KARFPQPEQLRD, PEQPFPQQDDGS, RRGQPFQPEQWL, QPFQPEQWLTLH, NGILGPEQPEQC, PEQPEQPIKIRI, VVNFPEQPESDK, RANHLNQPEQPF, GQPFQPEWLTLH, VVDPEQPQQDCT, QPEQPFVDQQDC, TRPDLEQPFPQP, FPEQPEDGILDI, HTYKYPEQPEQP, RRGQPFQPEWLT, GQPFWLTQPELH, RGQPFWQPEQLT, YPEQPEQPGSSE, ENPEQPEQIKIR, KARFPQPEQPFL, GQPFWQPEQPLT, GQPFWLTLQPEH, WLTLHFPEQPEG, WNFGQFPEQPED, GQPFWLQPEQPT, RGQPFWQPELTL, RGQPFWLTLQPE, SQPEQPFGMVNC, QPFWLTLHQPEQ, GQPFWQPELTLH, RGQPFWQPEQPF, QPEQPQQDCTLS, RRGEQPFPQPFW, QPFWLQPEQPTL, VLTQPEQPQQGF, RRGQPFWQPELT, YVLTPEQPFPQQ, QPFWLQPEQPFT, QPFWQPELTLHF, FWLTLHFPEQPE, RRGQPFWLTQPE, FPEQPESDKLKA, TYKYPEQPEQGS, GILGPEQPEQPF, DLEQPFPQPGYE, RDLYLEQPEQPF, QPFWLTLHFQPE, GQPFWQPEQPFL, VRRGQPFQPEQP, LHFPEQPEGRNY, VVRRGQPFWQPE, GQPFQPEQPFWL, GQPFWLQPETLH, GQFPEQPEDGIL, RGQPFWLQPEQT, RGQPFQPEQPFW, DWIPEQPFPQQD, RGQPFWLQPEQP, QPFQPEQPWLTL, GQPFWLTQPEQP, KLVVNFPEQPES, NFGQFPEQPEDG, RFPQPEQPLRDA, YKYPEQPEQGSS, GQPFQPEQPWLT, LNLEQPEQPFPF, LGPEQPEQPFCG, AGTKARFPQPEQ, YKYPEQPEQPGS, KRQPEQPFKLVA, FPEQPEGRNYEA, QFPEQPEDGILD, RRGPEQPFPQPF, RRGQPFWQPEQP, QPFWQPEQPFLT, TYKYPEQPEQPG, GSSEEREQPEQP, VRRGQPFWQPEL, QPFQPEQPFWLT, PEQPEQPGSSEE, VDWIQPEQPQQD, FGQFPEQPEDGI, EQPFPQPFWLTL, VNFPEQPESDKL, VRRGQPFWLQPE, LENPEQPEQIKI, GTKARFPQPEQL, RGQPFWLTQPEQ, NPEQPEQPFIKI, QPFWQPEQLTLH, RGQPFWLTQPEL, EQPEQPEVKVRM, VRRGEQPFPQPF |
