# Supplementary material for: Determination of B-Cell Epitopes in Patients with Celiac Disease: Peptide Microarrays
Source: PLoS One. 2016 Jan 29;11(1):e0147777. doi: 10.1371/journal.pone.0147777 (PMC4732949; doi:10.1371/journal.pone.0147777)
Supplement: S1 Text — (DOCX) [file pone.0147777.s007.docx]

**Supporting Information**

**S1 Text. Derivatization and Amino Acid Activation Solution Cocktails**

**Wafer Surface Derivatization**

Wafers were copiously washed with deionized water for 5 minutes and spin coated with a solution containing 1.25% (vol/vol) of 3-aminopropyltriethoxysilane (APTES) (Sigma-Aldrich) in N-methylpyrrolidone (NMP) (BDH Chemicals) and left at room temperature for 15 minutes. Curing of the wafers was done at 120°C for 60 minutes under N_2_ atmosphere. Wafers were then spin coated with a coupling solution containing 2 wt% of Fmoc-Gly-OH (AnaSpec), 2 wt% of hydroxybenzotriazole (HOBt) (AnaSpec), and 2 wt% of N,N'-diisopropylcarbodiimide (Sigma-Aldrich) in NMP and baked at 60°C for 5 minutes. This enables coupling of the activated carboxylic acid of Fmoc-glycine to the free amine present in APTES to form a covalent bond. Wafers were then rinsed with NMP and then capped with 50% (vol/vol) of acetic anhydride mixed with 50% of NMP to cap any remaining free APTES amines that had not been coupled. Wafers were stripped with acetone (BDH Chemicals) and isopropyl alcohol (BDH Chemicals). Fmoc protection of glycine was removed by spin coating the wafer with 5% (vol/vol) of piperidine (Sigma-Aldrich) in NMP and baking at 80°C for 300 seconds. The linker Fmoc-(PEG)_4_-COOH (AnaSpec) was then coupled to the wafer surface by spin coating a coupling solution containing 2 wt% of the linker, 2 wt% of HOBt, and 2 wt% of N,N'-diisopropylcarbodiimide in NMP and baked at 90°C for 120 seconds. Wafers were then rinsed with NMP and then capped with 50% (vol/vol) of acetic anhydride mixed with 50% of NMP to cap any remaining free amines. Wafers were stripped with acetone and isopropyl alcohol to complete the surface derivatization process.

**Amino Acid Activation Solution Cocktails**

Each amino acid activation solution cocktails was prepared as follows; First, 1 wt% of polymer (methyl methacrylate, MMA from Polysciences) was dissolved in NMP by sonication for 10 minutes. Then 2 wt% of Fmoc-amino acid (AnaSpec) was added to the solution, followed by addition of 2 wt% of HOBt (AnaSpec). Finally, 1 wt% of tetrazole thione was added to the cocktail. Amino acid activation solution cocktails were then filtered using a 0.05-µm filtration setup.

**Figure Legend for Supporting Data (S1 Text)**

**S1 Figure. Analysis of Peptide Purity.** Peptide LKWLDSFTEQ was synthesized and cleaved from the wafer substrate. Mass spectrometry shows the synthesized peptide mass, which is matched to the expected mass.
