# Supplementary material for: Determination of B-Cell Epitopes in Patients with Celiac Disease: Peptide Microarrays
Source: PLoS One. 2016 Jan 29;11(1):e0147777. doi: 10.1371/journal.pone.0147777 (PMC4732949; doi:10.1371/journal.pone.0147777)
Supplement: S2 Text — (DOCX) [file pone.0147777.s008.docx]

**Supporting Information**

**S2 Text. Peptide Purity and quality control**

**Analysis of Peptide Purity by Mass Spectrometry**

To verify the purity level of the synthesized peptide, after the synthesis process is completed, the synthesized peptide was cleaved from the wafer substrate. Then, the cleaved peptide was dissolved in 20% to 70% CH3CN/H2O in 1.75 minutes and loaded at 1.5 mL/min at 35°C in a Luna column (Phenomenex). Then, peptide mass was measured by mass spectrometry and matched the expected mass as shown in Supplemental Fig. 1.

**Fluorescein Quality Control**

After the synthesis process is completed, end-of-line fluorescein quality control is performed. The final amino acid in each peptide sequence is deprotected by base (10% [vol/vol] of piperidine in NMP) for 20 minutes and is coupled to a solution containing 1 wt% of 5(6)-carboxyfluorescein (AnaSpec), 2 wt% of N,N'-diisopropylcarbodiimide, and 2 wt% of hydroxybenzotriazole dissolved in NMP for 30 minutes. This is followed by successive washing steps with NMP (5 minutes), ethanol (5 minutes), mixture of 50 wt% of 1,2-ethylenediamine (Sigma-Aldrich) and 50 wt% of ethanol for 30 minutes, ethanol for 15 minutes, and isopropyl alcohol for 5 minutes. This process is used to analyze the individual coupling yield of each amino acid coupled in each step and also the step yield of each peptide sequence coupled. Sample data are as shown in Supplemental Fig. 2.
